# First Annual Commencement of the Medical Department of Niagara University

**Published:** 1886-05

**Authors:** 


### FIRST ANNUAL COMMENCEMENT OF THE MEDICAL DEPARTMENT OF NIAGARA UNIVERSITY.

### THE EXAMINATIONS—THE ORGANIZATION OF THE ALUMNI ASSOCIATION—THE CONVOCATION EXERCISES—THE HOOD AND GOWN—THE BANQUET.

We have for years most earnestly advocated a higher standard of medical education in our medical colleges, and therefore we have watched with great interest the progress of the effort to establish in this city a medical department, in which, by a provision of its corporate law, the high requirements which we and other journals of this country have demanded, and must, necessarily, be maintained.

Our readers well remember the organization of the Medical Department of Niagara University, three years ago, by twelve medical men in our city. Since then, there has been added to the faculty eight lecturers, some as assistants, and some as lecturers on special departments.

The college has given three full courses, and two spring courses. This year the winter, or regular term, was lengthened by adding six weeks to the course, and the spring term was omitted. The third term had drawn to a close, and examination week was at hand. For a week the faculty had examined the students, and at the end of that time the result was announced. Six out of the whole number examined for graduation were recommended by the Faculty to the Board of Examiners. Early Monday morning the Examiners arrived; they are: Dr. G. Doyle, Syracuse; Dr. Carroll, Rochester; Drs. Henry Flood and Ross, of Elmira; Drs. W. W. Potter, Hartwig, W. D. Granger, R L. Banta and C. W. Gould, of Buffalo, and organized by electing Dr. Banta, President, and assigned to each examiner a special branch for examination. The candidates were ordered before them at 10 A. M. They were examined for several hours. All the candidates passed with credit.

In the afternoon, at the Bishop's home, an *ad eundem* degree was conferred on the present faculty and lecturers of the college. Together with the graduates, they formed an alumni association to assist the college in all honest efforts to advance higher medical education. Of this association the officers are as follows: President, Dr. Wm. H. Heath; First Vice-President, Dr. R. B. Parks; 2d Vice-President, Dr. Murphy; Secretary, Dr. Geo. T. Lewis; Treasurer, Dr. Clark; Drs. Crego, Clark and A. Hill, Executive Board.

The exercises in the hall were the next in order. Many assembled at Association Hall in the evening to see the young graduates admitted, as of old, into the grade of the Doctorate. Bishop Ryan, as Chancellor of the University, addressed the meeting, being introduced by the president of the medical faculty, Dr. Cronyn. He told of the early beginnings of the University, the struggle for existence, but was glad to know that this college had not taken a second place anywhere. They require a preliminary examination; they require attendance at school, and a thorough examination before graduation; but all this is a help to the doctor, and in the distance he could see accorded this college the standing it deserved. He called attention to the hoods and gowns worn by the professors, lending dignity to their calling. They had seen fit to request the council to introduce the custom of Cambridge and Oxford here, and he was glad to be one to vote for its adoption. Too much could not be done to give these young men a high sense of their calling. They would also be hooded after the manner of the University, that they too might from henceforth be able to assume the insignia, won only by those who have been duly qualified to perform the high duties of their profession. He spoke of the Queen of England laying the corner stone of a medical school in London, finding herself among doctors with gown and hoods, lending true dignity to their noble calling. In conclusion he congratulated the medical faculty on the success attending their efforts, for in spite of their rigid entrance examination, the school numbered forty last year, and this was but the beginning. The young doctors then appeared before him one by one. They were presented by Dr. Tremaine, and the degree conferred by the Chancellor, the exercises being in Latin. At the end they were each hooded, a doctor's red hood being thrown over their heads. As the candidates came upon the stage, each was received by his friends with a burst of applause.

"There were many floral gifts. The young men were, without exception, fine-looking and doctor-like in their bearing."

The list of graduates is as follows: Dr. E. J. Murphy, Buffalo; Dr. R. B. Parks, Jamestown; Dr. Thomas Hill, Buffalo; Dr. Anthony Hill, Buffalo; Dr. George Wetherell, Toronto, Can.; Dr. George T. Lewis, Buffalo.

Prof. Clark, of the faculty, addressed the graduates. We are glad to be able to publish the remarks in full in the present number of the JOURNAL. The Rev. Mr. Bates, pastor of the Methodist Church, of Dunkirk, gave an excellent address, speaking extemporaneously. The effort was a scholarly one, and the speaker was frequently interrupted by applause. He called attention to the advance of medical science, and related many amusing anecdotes of medical treatment in various ages and countries. He called attention to the invaluable services rendered by the medical profession, and the poor return received. Their bravery, courage and faithfulness were all brought prominently before the audience, so that every doctor present must have felt the greatness of his calling. "It seems to me that what physicians need from the public is a little more evident gratitude. There is a rebound to gratitude that pays us again and again for all we give. It is the sweet music of the heart when its chords are swept by the breeze of kindness. There are some people that become as chronic in the search for doctors as in their disease, and usually, in the end, they dangle helplessly in the web of the patent medicine man. Nor is there more excuse for the discharge of our family physician because he has lost a patient in our family. We ought never employ a physician in whose hands we would not be willing to die."

"The public should never forget that a large part of the physician's ability and usefulness in the community is in its own hands. We generally get what we pay for, and it is only from Heaven that we can look for something for nothing. The young man that goes through the discipline of the schools and prepares himself for the practice of medicine by the widest as well as the most thorough special culture, who provides himself with the costly and necessary apparatus of his art, must feel that there is to be some adequate compensation for all his outlay. One reason why many a young physician never rises above mediocrity is because his best ambitions are not financially sustained. I know we say, "Where there is a will there is a way," but it remains equally true that we can sometimes make the will by making the way. It is to our own interest to throw around the young physician the best and most fruitful influences, and of these money is not the least. The tardiness with which a doctor's bills are often paid comports badly with the haste in which he was called. Could we but catch a glimpse of the uncollected accounts of a doctor's books, we would be shocked to know how many steal their physic. Probably this is one reason why the average of human life is not greater, for how can a thief thrive on stolen goods? "The wicked shall not live out half their days."

"With the public, then, rests a part of the physician's success, and, while his task is so responsible and his difficulties so great, let us prove to him that we are not ungrateful to him for his services nor forgetful of his needs. Let us receive his attentions, not as discharged duties but as sacrifices. Let us remember that we can give nothing that will fully remunerate him for the exchange of the quiet family hearth, the place where loved ones gather for the gloomy chamber of sickness and death. While we are permitted to share the sunshine and mirth of life, he lives under the cloud and in the storm. We truly say, what is life without health? What must be the physician's life bound to the body of death? All the diseases which Milton's imagination embodies in the Lager House, dog his footsteps and pluck at his door-bell.

"But there remains yet for the physician an unfailing inspiration in the fact that he practices not a human, but a divine art; for what is medicine but the application of those remedies that God has provided to ameliorate some of the effects of sin? All medicine is from God, and without Him it cannot exist or prosper. In the moral statutes of the Italian universities, we read: 'Our art, disunited from religion, is either impious or nothing.' The fate of the sick and the success of medicines are in the hands of Deity. Without Him, man can do nothing good for himself or his species."

At the close of this address, Dr. Henry Flood, of Elmira, N. Y., said he had been chosen by the Board of Examiners to make a few remarks to the public, as they were so well pleased with the result of their examinations. It certainly was a great triumph for the friends of higher medical education. They had subjected this class to as rigid an examination as it was possible, and all who were presented by the faculty had passed with credit.

The exercises were then over. Professors, students and friends repaired to the Genesee, where all, to the number of sixty, sat down to a banquet.

After the dinner, the regular order of toasts were responded to by the following gentlemen, in the order named:

Niagara University—Rev. Mr. Kavanaugh.
Medical Faculty—Dr. Cronyn.
Medical Profession—Dr. Briggs.
Medical Examiners—Dr. Henry Flood.
Legal Profession—Hon. John Congdon.
Clergy—Rev. Rufus Green.
Press—Rev. P. Cronin.
Adjunct Faculty—Dr. Crego.
Graduating Class—Dr. Wetherill.

The speeches were all good and to the point. Thus at an early hour the meeting adjourned, all having enjoyed themselves to the fullest extent.

